# Panel Practitioners and Hospital Beds

**Published:** 1913-02-01

**Authors:** 


### PANEL PRACTITIONERS AND HOSPITAL BEDS.

Deductions from premises, to some extent themselves conjectural, have been freely drawn as to the effects upon the voluntary hospitals of the operation of medical benefit on and after January 15. Now it is beginning to be possible to state with some degree of certainty what the first effects actually are; though it is as well to remark that the initial experience is not necessarily quite the same as that which will eventuate when the Act has been working for a few months. The prediction that there would be substantial relief in the over-crowded out-patient departments appears to be partly fulfilled. For the first week, we learn from inquiries at typical London hospitals, there was a considerable falling-off in numbers; but since then the diminution, though still existent, has been less marked. In the wards there was just at first also a lack of new patients. But now that the panel system has got to work there is as great a pressure on the available beds as usual, or even a greater.

As was also foreseen, the panel practitioners are sending large numbers of totally unsuitable cases with recommendations for treatment. It is impossible to blame them, for they are paid just the same whether they do their work or whether the hospitals do it for them; and it is only human nature that, harassed and overworked as they are, they should take somewhat limited and pessimistic views of their own capacities for treating panel patients. According to our information, hospital officers have had to refuse an enormous number of patients thus referred to them for in-patient treatment. The difficulty thus created is greatly intensified by the fact that there is, and can be, no hard-and-fast line between those cases which a practitioner ought to treat and those which he should hand over to hospitals. Thus a varicose ulcer is a troublesome chronic ailment, very disabling to its possessor, which will in nine cases out of ten heal quite readily if simple antiseptic cleanliness and complete rest can be secured for it. The panel doctor and the sickness benefit of the Insurance Act ought to be able to provide these requisites in most cases. Yet shoals of such cases have been flung at the general hospitals during the last fortnight. It is no doubt true that some very extensive and intractable cases of this disease can only be effectively relieved by skin-grafting; but it is distinctly untrue to suppose that this is called for except for a small minority. Even when skin-grafting is advisable, it might well be regarded as the duty of the panel doctor to perform such a very simple minor operation. This is one instance of the many sources of friction which the Act has created; and we mention it as a specimen, selected because we gather that it has been a particularly common instance during the last two weeks.

Another disease about which differences of opinion are inevitable is influenza, which is rather prevalent just now among the insured classes, though we have not heard of anything which can rightly be called an epidemic of it. Some cases of influenza undoubtedly begin with severe sudden symptoms which may suggest more serious diseases, such as pneumonia, gastric ulcer, and others. On the other hand, a good many cases of pneumonia counterfeit influenza very exactly for the first day or two; and there are other cases still in which broncho-pneumonia supervenes upon influenza. Then, again, it is quite possible to argue honestly and sincerely that any severe case of influenza is better off in a general hospital than in a labourer's dwelling, however "model" that may be in other respects. But it is also quite clear that the hospitals cannot possibly take in every case of influenza which the panel doctors would like to get rid of; nor is it their function to do so. Broadly speaking, therefore, it will be seen that the effects so far observed are very much those which we have all along predicted. When the hospitals loyally stated months ago that they were prepared to expect an increase of work in their in-patient departments, they did not bargain for every case which a harassed panel doctor might wish to foist on them.

